# AAA Rupture and Psoas Hematoma due to Type II Endoleak from Inferior Mesenteric Artery “Unusual” Collaterals

**DOI:** 10.1155/2017/8607437

**Published:** 2017-05-28

**Authors:** Panagiotis G. Theodoridis, Dimitrios N. Staramos, Nikolaos Ptochis, Ioannis A. Papailiou, Ilias Dodos, Nikolaos Iatrou, Anastasios G. Potouridis, Konstantinos Dervisis

**Affiliations:** ^1^Department of Vascular Surgery, “Konstantopouleio” General Hospital of Nea Ionia, Athens, Greece; ^2^Radiology Department, Division of Digital Subtraction Angiography, General Hospital of Athens “G. Gennimatas”, Athens, Greece; ^3^Radiology Department, Division of Interventional Radiology, “Konstantopouleio” General Hospital of Nea Ionia, Athens, Greece

## Abstract

Although endovascular aneurysm repair (EVAR) in the abdominal aorta has reduced the perioperative mortality when compared with open repair, the need for reintervention after complications such as endoleak may be presented in up to 20% of the cases. Type II endoleak from branch vessels is often benign but can potentially be associated with progressive abdominal aortic aneurysm growth and sac expansion. We present a rare case of a patient who presented with sac expansion and psoas hematoma due to Type II endoleak from “unusual” collaterals of IMA and was treated successfully with endoleak microembolization and percutaneous decompression of the hematoma.

## 1. Introduction

Endovascular repair (EVAR) of abdominal aortic aneurysms (AAAs) has been established as a successful alternative to open repair over the past 2 decades [1]. The early positive results in morbidity and mortality rate introduced by EVAR are balanced by the need for long-term surveillance and frequent reinterventions. Type II or branch vessel endoleaks (TIIELs) are often benign but can potentially be associated with progressive aggravation of the disease resulting in graft migration or sac expansion [2]. Aneurysm rupture after EVAR due to an isolated Type II endoleak is rare (<1% of all endoleaks). Furthermore, almost 30% of such ruptures occur with no sac expansion. There is a big debate around the proper management of these endoleaks. The scope of the current report is to present the diagnostic approach of an unusual case of a patient who presented with sac expansion due to a late TIIEL after EVAR and was treated endovascularly with embolization of a “peculiar” collateral branch of the IMA. Moreover, in the same patient, we emphasize the aggressive and immediate spooling of a symptomatic psoas hematoma aftermath of the aortic rupture.

## 2. Case Presentation

**Figure 1:**
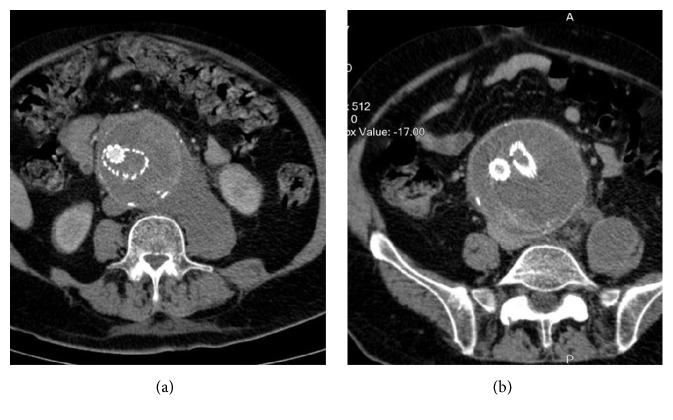
(a) Urgent CT reveals rupture of the aneurysm sac (a) in the arterial phase and (b) in the delayed phase without obvious endoleak.

**Figure 2:**
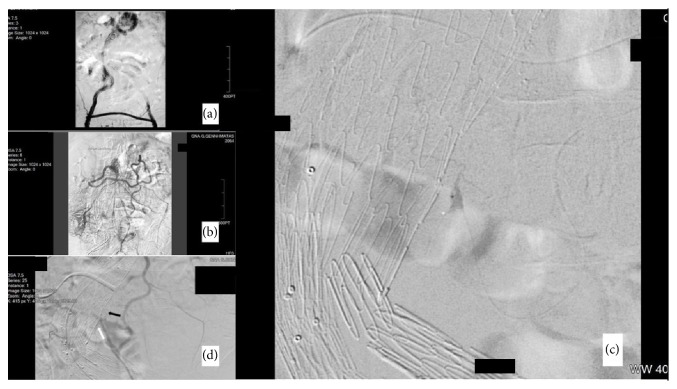
DSA angiography showing (a) antegrade flow through the aortounilateral stent graft and femorofemoral right to left bypass without Type I or III endoleaks, (b) the SMA and Riolano's collateral arch (c) infusion from the microcatheter which revealed blush from an “unusual” collateral, and (d) exclusion of endoleak (black arrow) using Onyx^R^ with protection of vital collaterals (white arrow).

A 71-year-old male patient was admitted to the Emergency Department of our hospital due to acute abdominal pain and drop consciousness. Urgent computed tomography (CT) revealed expansion of an 8.3 cm previously treated AAA sac without obvious endoleak (Figures 1(a) and 1(b)). After the initial fluids granting, the patient remained hemodynamically stable without need for blood transfusion or any intervention. The patient's medical history revealed hypertension, hypercholesterolemia, and EVAR for an asymptomatic 7.5 cm infrarenal AAA with a Medtronic Talent bifurcated stent graft (Medtronic, Dublin, Ireland) before ten years. The patient was operated on additionally twice after the initial EVAR. Initially, a Type Ib endoleak was found in a follow-up setting and a right leg extension was submitted in the external iliac artery. After that, the patient was operated on again because of a symptomatic Type III endoleak due to disconnection of the left leg from the main body. Then, an aortounilateral graft (Talent, Medtronic) was installed in the right side and a femorofemoral bypass was executed from the right common femoral artery (CFA) to the left CFA using a 6 mm e-PTFE standard wall graft. The left common iliac artery (CIA) was blocked with a plug (see Figure 2(a)).

Considering the increase in the aneurysmal sac and absence of visible endoleak in CT, we proceed in DSA control for further investigation. After exclusion of a Type I or III endoleak (Figure 2(a)), we followed the superior mesenteric artery (SMA) and Riolano's arch (Figure 2(b)) to visualize the inferior mesenteric artery (IMA). So, we used a microcatheter through the vertebral catheter in order to reach the ostium of the IMA. The angiography that followed revealed an uncommon inverted collateral branch of the IMA which was feeding the sack and caused the rupture (Figure 2(c)). We embolized it using Onyx with good result and rescue of the other vital branches (Figure 2(d)).

**Figure 3:**
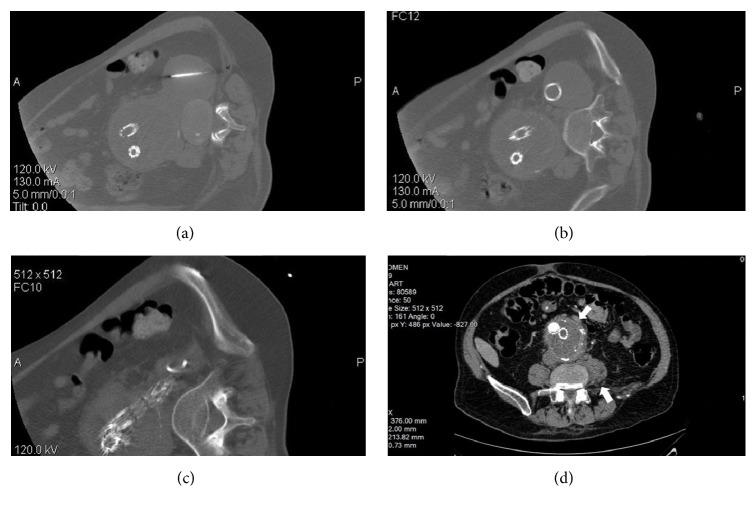
Drainage of hematoma under CT guidance: (a) guide trocar inserted from lumbar (b) pigtail in place drains the hematoma; (c) immediate shrinkage of hematoma cavity; (d) left iliopsoas muscle with almost normal shape (upward arrow) and reduction of remaining aneurysm sac (downward arrow) in a follow-up CT imaging four months later.

After the procedure, the patient remained stable without any clinical signs of sac growth. Unfortunately, in the third postprocedure day, he became sick with a fever of up to 39.5°C and pain in the left leg. The following CT showed growth of the hematoma which was connected through a fistula with the sac of the aneurysm. There was a speculation around the proper management of this complication. Taking into consideration the fact that the hematoma was about the sac, a steep fall of the pressure in the cavity might cause massive hemorrhage from the sac. On the other hand, the pressure from the hematoma may affect femoral nerve's function and can potentially be evolved into an organized abscess with unexpected effects. So, we decided to drain it percutaneously under CT guidance with satisfactory results. We inserted an 8 Fr locking pigtail catheter which evacuated the cavity immediately and remained for a month until the inflammation markers had fallen and the patient became stable without fever for two days (Figures 3(a)–3(c)). CT follow-up after four months showed shrinkage of both the sac and the hematoma (Figure 3(d)).

## 3. Discussion

EVAR for AAAs has been established as an alternative option superior to open repair in terms of aneurysm-related mortality and immediate morbidity. However, reintervention for complications such as endoleaks may be required in one-fifth of the cases. Several techniques (open, endovascular, or laparoscopic) have been described for the treatment of these complications [3].

Sac growth and rupture from undefined reasons are a common scenario for delayed endoleaks. In these cases, DSA control is a useful tool which can reveal TIIELs from unexpected feeding arteries. TIIELs are considered in most cases to be benign, since approximately one-third of them resolve spontaneously and they have no influence on mortality and rupture rate after EVAR. However, a significant number of patients require more than one procedure, and at 5 years, many patients who underwent embolization of a TIIEL continued to experience sac growth [4]. There is a big controversy around the proper management of TIIELs which, despite not being an urgent condition, can occasionally be associated with aortic rupture. In cases of transient TIIELs without sack growth or expansion, the preferred option is conservative management [5]. Nevertheless, there are a few persistent TIIELs where intervention is essential to protect patients from disastrous complications [6]. In cases where intervention is mandatory, percutaneous endovascular treatment with glue and/or coil embolization of the aneurysm sac, IMA, and lumbar branches via translumbar or transarterial approaches has been utilized to ablate such endoleaks. Diagnostic angiographic evaluation at the beginning of the procedure may reveal unexpected Type I or III endoleaks and is therefore recommended for all patients with TIIELs and sac growth. Nonetheless, these interventions for TIIELs with aneurysm sac growth do not appear to alter the progression of the disease and most patients display persistent/recurrent endoleak due to multiple anatomic mechanisms [7]. On the other hand, open surgical repair seems to have better results in the excision of the sac and the exclusion of the aneurysm but was associated with a higher incidence of severe complications [8].

The optimal method for observing aneurysm size and detecting endoleaks after EVAR remains undefined. Computed tomography angiography (CTA) has been widely accepted but can be limited by metal artifact from stents, which is exacerbated by embolization coils placed during the treatment of TIIELs. Despite the presence of streak artifact on CTA following coil embolization, it remains a useful diagnostic tool for following up patients. The presence of embolization coils does not prevent CTA measurement of aneurysm diameter and can detect recurrent endoleaks with a high degree of interobserver agreement [9]. Additionally, CTA is a useful material for detecting secondary complications after EVAR and planning therapeutic interventions. Psoas hematoma after AAA rupture is a possible complication with dangerous consequences in some cases. The percutaneous decompression of these hematomas is a high-risk selection but if performed successfully can relieve intractable pain and support functional restoration of the femoral nerve [10].

This case highlights (1) the occurrence of late TIIELs after EVAR and the possibility of being associated with sac rupture; (2) the diagnostic challenges associated with aortic rupture in cases of late TIIELs where CTA is not diagnostic and the need for super selective angiography of the IMA branches; (3) the need for draining of a symptomatic psoas hematoma irrespective of the possible risks.

## 4. Conclusion

TIIELs from unexpected collaterals after EVAR remain a potentially disastrous complication which may need careful surveillance and intervention in selective instances. Furthermore, secondary complications following EVAR are an existing threat which can increase the method's morbidity if not treated appropriately.
